# Association of Matrix Metalloproteinase1-1607 1G>2G Polymorphism and Lung Cancer Risk: An Update by Meta-Analysis

**DOI:** 10.31557/APJCP.2019.20.6.1841

**Published:** 2019

**Authors:** Yue Ma, Xi Yang, Yu-Ping Xie, Cheng Yi, Fen Zhao, Ying Huang

**Affiliations:** 1 *Department of Pathophysiology, West China School of Basic Medical Sciences and Forensic Medicine, *; 2 *Department of Medical Oncology, Cancer Center, West China Hospital, Sichuan University, *; 3 *Department of Oncology, Chengdu First People's Hospital, Chengdu 610041, Sichuan Province, China. *

**Keywords:** matrix metalloproteinase1 (MMP1), lung cancer, polymorphism, meta-analysis

## Abstract

**Objective:**

The association between matrix metalloproteinase1 (MMP1)-1607 1G>2G polymorphism and lung cancer risk is still inconclusive and inconsistent. We conducted a meta-analysis to estimate the potential relationship between MMP1-1607 1G>2G polymorphism and lung cancer risk.

**Methods:**

The comprehensive searches of the PubMed, Web of Science, Medline, CBM, CNKI, Weipu, and Wanfang databases, published up to Nov 10, 2018. Statistical analyses were performed with Review Manager 5.3 software.

**Results:**

A total of 14 relevant studies containing 6068 cases and 5860 controls were included in the study. The results indicated that MMP1-1607 1G>2G polymorphism was significantly associated with increased lung cancer risk under four models: 2G vs. 1G model (pooled OR = 1.19, 95% CI = 1.05-1.34, P < 0.0001); 2G/2G vs. 1G/1G (pooled OR = 1.34, 95% CI = 1.09-1.64, P = 0.003); 2G/2G vs. 1G/1G+1G/2G (pooled OR = 1.26, 95% CI = 1.06-1.49, P < 0.0001); 2G/2G+1G/2G vs. 1G/1G (pooled OR = 1.21, 95% CI = 1.05-1.40, P = 0.01). Subgroup analyses showed that there was a higher increase in smoking status under three models: 2G/2G vs. 1G/1G (pooled OR = 2.07, 95% CI = 1.14-3.77, P = 0.02); 2G/2G vs. 1G/1G+1G/2G (pooled OR = 1.71, 95% CI = 1.17-2.52, P = 0.006); 2G/2G+1G/2G vs. 1G/1G (pooled OR = 2.03, 95% CI = 1.14-3.62, P = 0.02). In addition, subgroup analyses by ethnicity further identified the significant association in Asians. Non-smoking population and ethnicity among Caucasian had no relationship with lung cancer susceptibility in four models.

**Conclusion:**

Our study suggested that MMP1-1607 1G>2G polymorphism was a risk factor for developing lung cancer risk.

## Introduction

Lung cancer (LC), as the most common cancer worldwide, accounted for 2.1 million new LC cases and 1.8 million deaths predicted in 2018 (McIntyre and Ganti, 2017; Bray et al., 2018). Now, the mechanism of LC still remains unclear. The major risk factors are involved in environmental pollution, genetic susceptibility, tobacco smoking and diet (Malhotra et al., 2016). Although these factors have been demonstrated to promote the incidence of LC, not all individuals exposed in the same environment develop the disease. This also indicates that other causes, such as genetic variants, may lead to the development of LC. In recent years, numerous published studies have focused on the association of genetic variants with LC susceptibility, and among which, the matrix metalloproteinase (MMP) gene has been extensively studied.

MMP is a family of zinc-dependent proteolytic enzymes that are able to degrade the components of the extracellular matrix (ECM) including basement membranes and collagen (Nelson et al., 2000). MMP1, one member of the MMP, performs a critical function in degrading the interstitial collagen types I, II, III and mediating pathways of angiogenesis (Vincenti et al., 1996). The expression level of MMP1 can be affected by a functional single nucleotide polymorphism (MMP1-1607 1G>2G). 1607 1G>2G in the MMP1 gene contains a single-guanine insertion/deletion 2G to 1G polymorphism located at the MMP1 promoter region (Hu et al., 2012). The 2G allele has been associated with higher transcriptional activity due to creating a transcription factor binding site (Rutter et al., 1998). Despite the fact that increasing studies on the association of genetic risk of 1607 1G>2G polymorphism in the MMP1 gene for LC have been published in the past decades, the results remain inconsistent and controversial. To providing a more comprehensive conclusion, we performed a meta-analysis based on 14 eligible case-control studies to evaluate the associations between MMP1-1607 1G>2G polymorphism and LC risk.

## Methods and Materials

*Literature search and study selection criteria*

The PubMed, Web of Science, Medline, Chinese biomedical (CBM), China national knowledge infrastructure (CNKI), Weipu, and Wanfang databases were searched to identify all articles by using the following keywords (last search was updated on Nov 10, 2018): “MMP1 or matrix metalloproteinase1” and “lung tumor or lung cancer or lung neoplasm or lung carcinoma” and “polymorphism or variant or mutation”. The inclusion criteria were listed as follows: (1) English or Chinese publications; (2) investigating the 1607 1G>2G polymorphism in MMP1 gene and LC risk; (3) case-control studies; (4) available data for calculating an odds ratio (OR) with corresponding 95% confidence interval (95% CI) and a P value. The studies with overlapping cases or controls were excluded in the meta-analysis. 

*Data extraction*

Data information was independently extracted by two reviewers (Ma and Xie) for compliance with the above inclusion criteria. Any discrepancies were carefully checked by a third author and resolved by discussion. The summary data information included: first author, the year of publication, country of the study, ethnicity, total genotypes of cases and controls, study design defined as population-based case-control study (PCC) and hospital-based case-control study (HCC), and genotyping method. 

*Statistical analysis*

Effect size was expressed as ORs with their 95% CI to assess the strength of the potential association between MMP1-1607 1G>2G polymorphism and LC risk. We estimated the risk under an allele model (2G vs. 1G), a homozygous model (2G/2G vs. 1G/1G), a recessive model (2G/2G vs. 1G/1G+1G/2G) and a dominant model (2G/2G+1G/2G vs. 1G/1G), respectively. According to the heterogeneity, a fixed-effects model or a random-effects model was used to calculate pooled ORs. The study heterogeneity assumption was tested using a χ^2^-based Q test and a P value of less than 0.05 was considered representative of statistically significant. The fixed–effects model was conducted to calculate the pooled ORs if no significant heterogeneity was observed (Q test results with P > 0.10); otherwise, the random-effects model was conducted. We performed subgroup analyses on smoking status to evaluate smoking status-specific including smoker group and non-smoker group. Moreover, subgroup analyses were also performed based on ethnicity. Different ethnicities were categorized into Caucasians and Asians.

Sensitivity analyses were performed on individual studies to assess the stability of the results. Begg’s funnel plots and Egger’s tests (P < 0.05 considered statistical significance) were used to investigate the possible publication bias (Begg and Mazumdar, 1994; Egger et al., 1997). HWE was measured based on an Internet program (P > 0.05). All statistical analyses were done with RevMan software (version5.3; Cochrane Collaboration).

## Results

*Included study characteristics*

**Figure 1:**
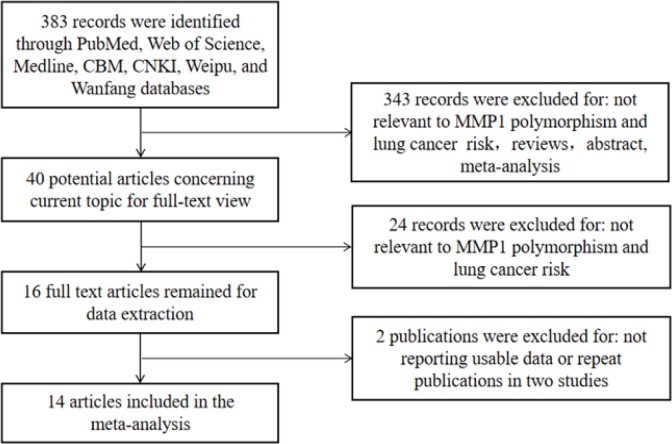
Flow Diagram of Included/Excluded Studies

As illustrated in Figure 1, the initial literature search yielded 383 studies for detailed review. Forty potential relevant studies were included to assess in detail after reading the abstracts. Twenty-four additional articles were excluded for being irrelevant to LC risk and MMP1-1607 1G>2G polymorphism. In addition, two studies were also excluded for the repeat data (Su et al., 2005; Fang et al., 2006). Finally, the remaining 14 studies with 6,068 cases and 5,860 controls fulfilled our inclusion criteria. Table 1 listed the main characteristics of the studies identified for MMP1-1607 1G>2G polymorphism in the meta-analysis. Among 14 studies in the present meta-analysis, there were 11 English articles (Biondi et al., 2000; Zhu et al., 2001; Fang et al., 2005; Schabath et al., 2005; Su et al., 2005; Gonzalez-Arriaga et al., 2008; Klinchid et al., 2009; Hart et al., 2011; Liu et al., 2011; Fakhoury et al., 2012; Shen et al., 2018) and 3 Chinese articles (Zhang et al., 2006; Cheng, 2007; Wei et al., 2007). Moreover, 3 articles were performed in smoking status study (Zhu et al., 2001; Fang et al., 2005; Wei et al., 2007). Of those, 7 articles were conducted on Caucasian population (Biondi et al., 2000; Zhu et al., 2001; Schabath et al., 2005; Su et al., 2006; Gonzalez-Arriaga et al., 2008; Hart et al., 2011; Fakhoury et al., 2012) and 7 on Asian population (Fang et al., 2005; Zhang et al., 2006; Cheng, 2007; Wei et al., 2007; Klinchid et al., 2009; Liu et al., 2011; Shen et al., 2018). Genotypes and allele distributions of the studies were presented in Table 2. 

*Quantitative meta-analysis results*

**Figure 2:**
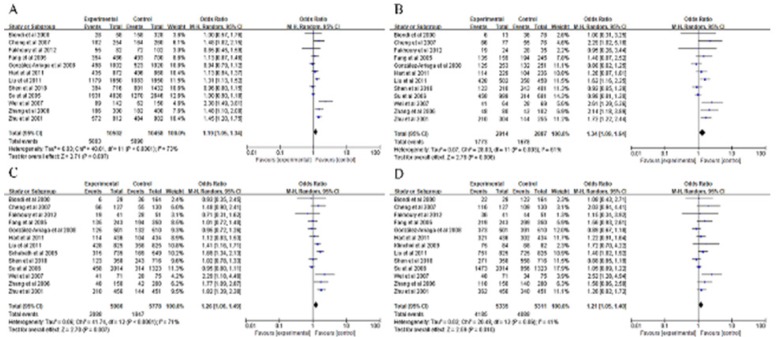
Meta-Analysis for the Association between Lung Cancer Risk and the MMP1-1607 1G>2G Polymorphism (A the allele model 2G vs. 1G. B the homozygous model 2G/2G vs. 1G/1G. C the recessive model: 2G/2G vs. 1G/1G +1G/2G. D the dominant model: 2G/2G +1G/2G vs. 1G/1G). CI, confidence interval; OR, odds ratio

The stratified summary results were shown in Table 3. In the allele model (2G vs. 1G), the overall pooled effect suggested that the 2G allele carrier may have 19% increased LC risk compared with 1G the allele carrier (pooled OR = 1.19, 95% CI = 1.05-1.34, P < 0.0001) (Figure 2A). In the homozygous model and the recessive model, the 2G/2G homozygote had a significant association with increased LC risk, compared with the 1G/1G homozygote (pooled OR = 1.34, 95% CI = 1.09-1.64, P = 0.003) (Figure 2B) and 1G/1G+1G/2G genotype (pooled OR = 1.26, 95% CI = 1.06-1.49, P < 0.0001) (Figure 2C). Similarly, it was also indicated that the 2G/2G+1G/2G genotype was associated with a significantly increased LC risk, compared with the 1G/1G homozygote (pooled OR = 1.21, 95% CI = 1.05-1.40, P = 0.01) (Figure 2D) in the dominant model. 

**Figure 3:**
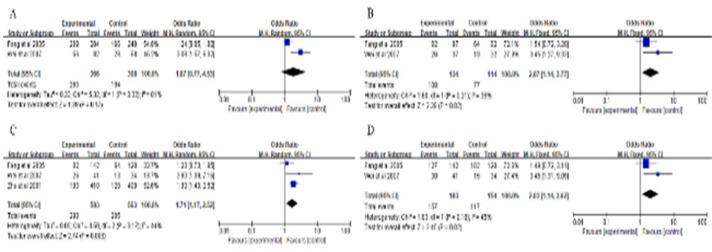
Meta-Analysis with a Fixed-Effects Model for the Association between Lung Cancer Risk and the MMP1-1607 1G>2G Polymorphism in Smoking Studies with Different Genotypes (A the allele model 2G vs. 1G. B the homozygous model 2G/2G vs. 1G/1G. C the recessive model: 2G/2G vs. 1G/1G +1G/2G. D the dominant model: 2G/2G +1G/2G vs. 1G/1G). CI, confidence interval; OR, odds ratio

**Figure 4:**
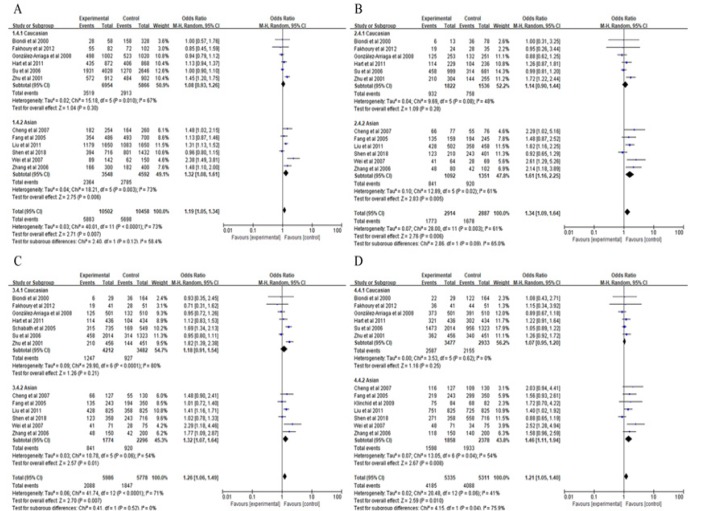
Meta-Analysis with a Fixed-Effects Model for the Association between Lung Cancer Risk and the MMP1-1607 1G>2G Polymorphism in Subgroup Analysis by Ethnicity (A the allele model 2G vs. 1G. B the homozygous model 2G/2G vs. 1G/1G. C the recessive model: 2G/2G vs. 1G/1G +1G/2G. D the dominant model: 2G/2G +1G/2G vs. 1G/1G). CI, confidence interval; OR, odds ratio

We conducted a subgroup analysis in smoking status and another subgroup in ethnic group under various genetic models (Figure 3). Significantly elevated risks were observed among smokers under there models (the homozygous model, pooled OR = 2.07, 95% CI = 1.14-3.77, P = 0.02; the recessive model, pooled OR = 1.71, 95% CI = 1.17-2.52, P = 0.006; the dominant model pooled OR = 2.03, 95% CI = 1.14-3.62, P = 0.02), but not under an allele model (pooled OR = 1.87, 95% CI = 0.77-4.53, P = 0.17). Thus, MMP1-1607 1G>2G polymorphism in smokers may have a higher increase LC risk under the homozygous model, the recessive model and the dominant model. In ethnicity subgroup analyses (Figure 4), significantly increased risks were observed in Asian group under four models: 2G vs. 1G, pooled OR = 1.32, 95% CI = 1.08-1.61, P = 0.006 (Figure 4A); 2G/2G vs. 1G/1G, pooled OR = 1.61, 95% CI = 1.16-2.25, P = 0.005 (Figure 4B); 2G/2G vs. 2G1G+1G/1G, pooled OR = 1.32, 95% CI = 1.07-1.64, P = 0.01 (Figure 4C); 2G/2G+1G/2G vs. 1G/1G, pooled OR = 1.46 (Figure 4D), 95% CI = 1.11-1.94, P = 0.008. However, no significant association was observed in non-smoker group and Caucasians under four genetic models. Thus, MMP1-1607 1G>2G polymorphism increased LC risk in Asians.

*Sensitivity analysis*

In the present meta-analysis, we carried out a sensitivity analysis through sequential omission of a single study to evaluate the stability of the results. The results indicated that no individual study dominantly affected the overall ORs. Statistically similar outcomes were obtained in total studies (all P > 0.05), conforming the stable results (data not shown).

*Publication bias*

**Figure 5:**
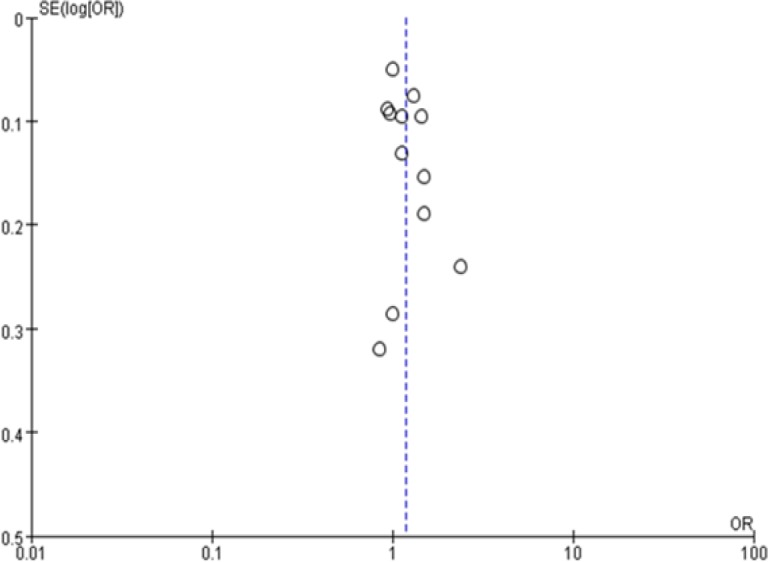
Begg’s Funnel Plot for Publication Bias in Selection of Studies on the MMP1-1607 1G>2G Polymorphism (2G vs. 1G). OR, odds ratio

We used the funnel plots and Egger’s tests to assess the publication bias. The graphical funnel plot of MMP1-1607 1G>2G polymorphism under 2G vs. 1G, the allele model, did not show any evidence of obvious asymmetry (Figure 5). The Egger’s tests showed that there were no publication bias (all P > 0.05) (data not shown).

**Table 1:** Characteristics of the Studies Included in Meta-Analysis

First author	Year	Country	Ethnicity	Study design	No.	Genotyping
					(Cases/Controls)	method
Biondi et al., (2000)	2000	Italy	Caucasian	NA	29/164	NA
Cheng et al., (2007)	2007	China	Asian	HCC	125/130	PCR-RFLP
Fakhoury et al., (2012)	2012	Lebanon	Asian	PCC	41/51	PCR-RFLP
Fang et al., (2005)	2005	China	Asian	PCC	243/350	PCR-RFLP
González-Arriaga et al., (2008)	2008	Spain	Caucasian	HCC	501/510	PCR-RFLP
Hart et al., (2011)	2011	Norway	Caucasian	PCC	436/434	TaqMan real-time PCR
Klinchid et al., (2009)	2009	Thailand	Asian	HCC	84/82	PCR-RFLP
Liu et al., (2011)	2011	China	Asian	PCC	825/825	PCR-RFLP
Schabath et al., (2005)	2005	USA	Caucasian	HCC	735/549	NA
Shen et al., (2018)	2018	China	Asian	HCC	358/716	PCR-RFLP
Su et al., (2006)	2006	USA	Caucasian	PCC	2014/1323	TaqMan
Wei et al., (2007)	2007	China	Asian	HCC	71/75	PCR-RFLP
Zhang et al., (2006)	2006	China	Asian	PCC	150/200	PCR-RFLP
Zhu et al., (2001)	2001	USA	Caucasian	PCC	456/451	PCR-RFLP

PCR, polymerase chain reaction; PCR-RFLP, polymerase chain reaction-restriction fragment length polymorphism; Population-based case-control study (PCC); Hospital-based case-control study (HCC).

**Table 2:** Distribution of MMP1-1607 1G>2G Genotype and Allele among Lung Cancers and Controls

First Author	Cases (n)			Controls (n)			Cases (n)		Controls (n)		HWEa for control	
	1G/1G	1G/2G	2G/2G	1G/1G	1G/2G	2G/2G	1G	2G	1G	2G	P	χ2
Biondi et al., (2000)	7	16	6	42	86	36	30	28	170	158	0.52	0.413
Cheng et al., (2007)	11	50	66	21	54	55	72	182	96	164	0.217	1.523
Fakhoury et al., (2012)	5	17	19	7	16	28	27	55	30	72	0.081	3.047
Fang et al., (2005)	24	84	135	51	105	194	132	354	207	493	0	27.395
González-Arriaga et al., (2008)	128	248	125	119	259	132	504	498	497	523	0.712	0.136
Hart et al., (2011)	115	207	114	132	198	104	437	435	462	406	0.081	3.044
Klinchid et al., (2009)	9	NA	75^a^	14	NA	68^a^	NA	NA	NA	NA	NA	NA
Liu et al., (2011)	74	323	428	100	367	358	471	1179	567	1083	0.691	0.158
Schabath et al., (2005)	NA	420^b^	315	NA	380^b^	169	NA	NA	NA	NA	NA	NA
Shen et al., (2018)	87	148	123	158	315	243	322	394	631	801	0.004	8.28
Su et al., (2006)	541	1015	458	367	642	314	2097	1931	1376	1270	0.31	1.031
Wei et al., (2007)	23	7	41	41	6	28	53	89	88	62	0	52.297
Zhang et al., (2006)	32	70	48	60	98	42	134	166	218	182	0.865	0.029
Zhu et al., (2001)	94	152	210	111	196	144	340	572	418	484	0.007	7.176

HWE^a^, Hardy-Weinberg equilibrium; ^a^Numbers of 1G/2G + 2G/2G; ^b^Numbers of 1G/1G + 1G/2G; NA, not available

**Table 3:** The Meta-Analysis for the Associations between MMP1-1607 1G>2G Polymorphism and Risk of Lung Cancer

Variables (No. studies)	2G vs. 1G		2G/2G vs. 1G/1G		2G/2G vs. 1G/1G+1G/2G		2G/2G+1G/2G vs. 1G/1G	
	OR (95% CI)	P	OR (95% CI)	P	OR (95% CI)	P	OR (95% CI)	P
Total (14)	**1.19 [1.05, 1.34]**	**0.01** ^a^	**1.34 [1.09, 1.64]**	**0.01** ^a^	**1.26 [1.06, 1.49]**	**0.01** ^b^	**1.21 [1.05, 1.40]**	**0.01** ^c^
Subgroup by smoker status								
Smoker(3)	1.87 [0.77, 4.53]	0.17	**2.07 [1.14, 3.77]**	**0.02**	**1.71 [1.17, 2.52]**	**< 0.01**	**2.03 [1.14, 3.62]**	**0.02**
Nonsmoker(3)	1.15 [0.81, 1.61]	0.44	1.48 [0.79, 2.80]	0.22	1.03 [0.69, 1.54]	0.87	1.57 [0.85, 2.87]	0.15
Subgroup by study design								
Caucasian(7)	1.08 [0.93, 1.26]	0.3	1.14 [0.90, 1.44]	0.28	1.18 [0.91, 1.54]	0.21	1.07 [0.95, 1.20]	0.25
Asian(7)	**1.32 [1.08, 1.61]**	**< 0.01**	**1.61 [1.16, 2.25]**	**< 0.01**	**1.32 [1.07, 1.64]**	**0.01**	**1.46 [1.11, 1.94]**	**< 0.01**

## Discussion

Our meta-analysis including 14 studies with 6068 cases and 5860 controls suggested that MMP1-1607 1G>2G polymorphism was associated LC risk under four genetic models: 2G vs. 1G model (pooled OR = 1.19, 95% CI = 1.05-1.34); 2G/2G vs. 1G/1G (pooled OR = 1.34, 95% CI = 1.09-1.64); 2G/2G vs. 1G/1G+1G/2G (pooled OR = 1.26, 95% CI = 1.06-1.49); 2G/2G+1G/2G vs. 1G/1G (pooled OR = 1.21, 95% CI = 1.05-1.40). It suggested that MMP1-1607 1G>2G polymorphism was significantly associated with increased LC risk.

MMP1, a member of MMP in degrading the interstitial collagen types I, II, III, is constitutively expressed in normal physiologic conditions at a low level, while in pathologic conditions its expression may increase remarkably especially in cancer. MMP1-1607 1G>2G polymorphism may have impacts on the susceptibility to cancer risk by modulating MMP1 expression levels. A recent meta-analysis study explored the association between MMP1 polymorphism and cancer susceptibility (Han et al., 2014). It has been proved that MMP1-1607 1G>2G polymorphism elevated risk was found regarding lung cancer, colorectal cancer, head and neck cancer and bladder cancer. Xiao et al., (2012) also have reported that 1607 1G>2G polymorphism in the MMP1 increased LC risk in Asians. Our results supported the conception that MMP1-1607 1G>2G polymorphism was a genetic susceptibility risk factor of LC.

It is well recognized that smoking cigarettes can cause LC. Many carcinogens are present in cigarette smoke, some of which can stimulate the growth of lung cancer cells (Wen et al., 2016). Interactions with some gene polymorphisms and smoking are associated with increased risk of LC (Herbst et al., 2008). In the subgroup, we analyzed the smoking status concluding 3 studies to evaluate relationship between smoking and MMP1-1607 1G>2G for LC. Strongly significant associations were observed in smokers under three genetic models: 2G/2G vs. 1G/1G (pooled OR = 2.07, 95% CI = 1.14-3.77); 2G/2G vs. 1G/1G+1G/2G (pooled OR = 1.71, 95% CI = 1.17-2.52); 2G/2G+1G/2G vs. 1G/1G (pooled OR = 2.03, 95% CI = 1.14-3.62). These results indicated that there were significant associations with higher LC risks under the homozygous model, the recessive model, and the dominant model. To the best of our knowledge, this is the first meta-analysis to explore the association between MMP1-1607 1G>2G polymorphism and LC for smoking population, involving 593 cancer cases and 563 controls. Zhu et al. investigated that the 2G allele of the MMP1 promoter single-nucleotide polymorphism had been associated with increased LC susceptibility in Caucasians, especially among smokers (Zhu et al., 2001). However, in our subgroup analyses by ethnicity, the association was still obvious in Asians, but there was no association in Caucasians. Our results were consistent with previous meta-analyses (Xiao et al., 2012; Lu et al., 2015). The total sample sizes of Caucasians might be limited and the discrepancies between Asians and Caucasians might be due to some factors, such as complicated environment and various genetic backgrounds. In different populations, the inconsistent result still demonstrated the importance on assessing genetic effects on disease development and progression.

There were some advantages in our meta-analysis compared with previous studies. First of all, updated statistics for the MMP1-1607 1G>2G polymorphism and LC risk were based on a larger size of cases and control subjects. Then, the symmetrical funnel plots and Egger’s tests showed that there were no publication bias, suggesting the unbiased pooled results. Finally, we investigated the smoking status to analyze the connection between smoking and 1607 1G>2G polymorphism of MMP1 for LC risk. However, this meta-analysis had several limitations that should be addressed. Firstly, the eligible studies were written in English or Chinese, which may cause potential bias for missing publications. Secondly, the insufficient genotype data in the studies of Klinchid et al. and Schabath et al. may affect our results. Thirdly, the quantity of objectives were limited especially in smoking subgroup. Consequently, the total samples require to be expanded. Fourthly, despite subgroups being conducted by smoking and ethnicity, there were still a few potential factors that may be ignored, such as age, gender and family history. Thus, further studies should be conducted considering the gene-environment interactions. 

In conclusion, the present meta-analysis showed MMP1-1607 1G>2G polymorphism was a risk factor for developing LC risk. Subgroup analyses based on smoking suggested a higher association between MMP1-1607 1G>2G polymorphism and LC risk under the homozygous model 2G/2G vs. 1G/1G, the recessive model 2G/2G vs. 1G/1G+1G/2G, the dominant model 2G/2G+1G/2G vs. 1G/1G. And there was a significant association between the MMP1-1607 1G>2G polymorphism and LC risk in Asians. Moreover, future studies are still required with a larger population and detailed environmental backgrounds.
